# Study on Identification and Prevalence of Ixodid Ticks Genera Infestation in Cattle in the Case of Areka District, Wolaita Zone, and Southern Ethiopia

**DOI:** 10.1155/2023/6389473

**Published:** 2023-02-27

**Authors:** Gizachew Fentahun, Fanuel Bizuayehu, Teshager Dubie

**Affiliations:** College of Veterinary Medicine, Samara University, P.O. Box 132 Samara, Ethiopia

## Abstract

A cross-sectional study was conducted from November 2021 to July 2022 to determine the prevalence and identify major ixodid ticks of cattle and associated risk factors in the case of Areka District, Woliata Zone, and Southern Ethiopia. Standard physical and direct stereomicroscopy techniques were employed for identification of tick genera. Descriptive statistics and chi-squared test analyses were used for data analysis and *P* < 0.05 was considered as significant. During the study period, a sample of 384 local breed cattle's were taken by random selection and a total of 683 adult ixodid ticks were collected from different body parts of infested animals. Among 384 examined animals, 275 (71.6%; 95% CI: 62.8–80.4) animals were infested with one or more ixodid tick genera. In current study, the major ixodid tick genera infesting cattle were *Ambylomma* (32.2%), *Rhipicephalus (Boophilus)* (30%), *Haylomma* (16.8%), and *Rhipicephalus* (21%), and most of the genera preferred dewlap and sternum body part of animal for attachment. Out of 184 male and 200 female cattle examined, 144 males (78.3%) and 131 females (65.5%) were positive for one or more adult ixodid tick genera. The difference also found statically significant (*P* < 0.05). The overall prevalence of hard tick's infestation was statistically significant (*P* < 0.05) across the age, origin, and body condition of cattle. In conclusion, the high level of prevalence of hard ticks infestation in the present study represent the most important problems of cattle and detrimental to production. According to this finding, it is recommended that owners who keep cattle should practice good management and regular deworming using acaricides and it is also important to create awareness among livestock owners about the veterinary importance of ticks for the integrated tick control.

## 1. Introduction

Ethiopia has the largest livestock number in Africa about 59.5 million cattle, 30.7 million sheep, 30.2 million goats, 2.16 million horses, 8.44 million donkeys, 0.41 million mules, 1.21 million camels, and about 56.3 million poultry [1]. Ethiopian livestock system are important sources of income for the agricultural communities as sources of animal protein, providing of meat and milk consumption, and also the major sources of foreign currency through skin and meat export [2]. However, even though the country has endowed with large and varied livestock populations particularly in cattle population; the livestock sector is challenged by different constraints like shortage of feed, disease, poor policy, inadequate veterinary service, and poor infrastructures resulted stumbling block to the potential of livestock industry [3].

Among diseases, parasites, particularly tick cause significant loss of livestock production. Ticks are external parasites of both domestic and wild animal and cause detrimental effects on their hosts through puncture, burrow, discomfort, annoyance, weight loss, loss of condition, reduction in milk and meat production, irritation of the skin, and predispose to infection [4]. Ticks transmit a wide variety of veterinary diseases, such as protozoa, bacteria, fungi, and viruses, which infect domestic livestock and wild animals as well as humans in most regions of the world [5].

Ticks have worldwide distribution and have a preference humid and temperate atmosphere and typically attach to the site of legs, neck, and under-abdomen of their host body and suck host blood during their lengthy attachment period (7–14 days), which may be extended depending on the tick genus and unique host association [6]. In Ethiopia, hard ticks are found in all agro ecological zones of the country and different tick genera are widely distributed and a number of researchers reported the distribution and abundance of tick species in different part of the country [7]. The isolated ixodid ticks in the country, which belong to genus level are *Amblyomma*, *Rhipicephalus*, *Haemaphysalis*, *Rhipicephalus (Boophilus)*, and *Hyalomma* [8].

Tick infestation causes the decreased quality of skin (hide) up to 20–30% and causes severe anemia, weakness, and immune suppression in the infested animals [9]. It is estimated that 80% of the global cattle population is at risk of ticks and tick-borne diseases (TBDs) resulting in annual global production losses of ∼USD 22 billion to 30 billion, with the majority of these losses occurring in developing countries [6]. Economic losses attributable to ticks in cattle are caused either by damage to hides and udders, or through mortality or debility caused by TBDs. These substantial losses adversely impact the livelihood of poor-resource farmers, due to reduced milk yields, live weight gains in cattle, and damage to hides [10]. Even though a research conducted in different part of the country, there is a dearth of information on prevalence determination and identification of hard tick genus in study area, particularly in Areka District. Therefore, the present study was carried out with the objective of estimating prevalence and identification of hard tick at genus level and its associated risk factors in the case of Areka District, Wolaita Zone, and Southern Ethiopia.

## 2. Materials and Methods

### 2.1. Study Area

The study was conducted in Areka District, Wolaita Zone, Southern Nations, Nationalities, and Peoples' Regions (SNNPRs) of Ethiopia. Areka is located 350 km from capital city of Ethiopia. It is also located 1100–2300 m above sea level, 6°40′;N latitude and 37°50′;E longitude. The study area has mean annual temperature of 22°C and receives average rainfall of 1123.15 mm. It is bordered on the South by West Boloso Sore District, North by Sodo Zuria District, East by Gununo District, and West by Hadiya District. The number of cattle in Areka District is estimated to be 80,822 [11].

### 2.2. Study Animals

Cattle kept under an extensive production system and having different age, sex, origin, and body conditions were included in the study. The age of cattle were classified as young, adult, and old [12] and estimated based own owner's information and using the eruption pattern of teeth. Moreover body conditions of the cattle were classified as poor, medium, and good [13] and determined by palpating and observing the vertebrae of loin regions.

### 2.3. Study Design

A cross-sectional study design was conducted to identify and determine the prevalence of ixodid tick in cattle in Areka District of Wolaita Zone from November, 2021 to July, 2022.

### 2.4. Sampling Method and Sample Size Determination

The sample size was calculated using 95% confidence level, 50% expected prevalence, and 5% desired absolute precision as described by Thrusfeld [14], since there is no previous study conducted in this study area. Accordingly, formula is as follows:

(1) $$\begin{array}{c} N=\frac{Z^{2}\times P_{\exp}\;\left(1-P_{\exp}\right)}{d^{2}}, \end{array}$$

where *N* = require sampling size, *P*_exp_ = expected prevalence, *d*^2^ = desired absolute precision, and *Z* = constant value from normal distribution table at a given confidence level (1.96). Therefore, 384 cattle were selected using simple random sampling method. The study sites were selected based cattle population, convenience, and transportation. Accordingly, five kebeles namely, Woyibo, Korke, Legamo, Tokiso, and Adibincho, were involved in the study.

### 2.5. Sample Collection and Transportation

The entire body surface of the host was inspected for ticks. After fully restraining the animal, all visible adult ticks were detached carefully with the help of blunt steel forceps with great care so as not to lose the mouthparts of the ticks. Ticks were collected on different body parts, particularly on the animal's dewlap, udder, scrotum sternum, and other body parts (anal, tail, ear, and leg). Ticks from each animal were collected and preserved in universal bottles containing 70% ethanol and labeled with required information like date of collection, age, sex, and origin and body condition scores before transportation to parasitology laboratory for identification. The labeled sterile bottle containing tick samples were transported to Wolaita Sodo Regional Veterinary Parasitology Laboratory for morphologically identification ixodid tick at genus level using stereomicroscope. The tick genera naming was made according to the keys and descriptions given by [8]). The taxonomic identification of ixodid tick was based on respiratory stigma, coxae, base of the gnathosoma, mouth parts, scutum, festoons, position, and presence or absence of punctuations on the body.

### 2.6. Data Management and Statistical Analysis

The data obtained in this study were recorded in the Microsoft-Excel spreadsheet 2007 and coded. Then, the coded data were being import into the SPSS version 16 software. Descriptive statistics were used to determine the prevalence and identification of ixodid tick at the genus level. Correlation between number of tick-infested animals for each risk factor (sex, body condition, age, and origin) of cattle was subjected to chi-squared test (*χ*^2^) to determine the association.

## 3. Results

### 3.1. Overall Prevalence of Tick Infestation and Associated Risk Factors

A sample of 384 local breed cattle were taken by random selection and 683 adult ixodid ticks were collected from different body parts of infested animals. Out of 384 cattle examined for the presence of ixodid ticks, 275 (71.6%; 95% CI: 62.8–80.4) were found to be infested with varying numbers of tick genera (Table 1). Higher tick prevalence was recorded in Korke Kebele (79.1%), whereas the least prevalence was recorded in Woyibo Kebele (53.2%) with statistically significant difference (*P* < 0.05). The occurrence of tick infestation across sex, age, and body condition of animals was also found to be significantly different (*P* < 0.05) with higher prevalence in sex of male (78.3%), old age (82.3%), and poor body condition (87.2%) of animal and the lower prevalence in sex of female (65.5%), young age (59.8%), and good body condition (57.5%) of animal (Table 1).

### 3.2. Ixodid Ticks Burden, Sex Ratio, and Genera Identification

A total of 683 adult ticks were collected from different body parts of infested animals in the study sites. From the total of tick collected genera, male accounts 74.8% (511) and female 25.2% (172; Table 2). This shows that infestation by male tick was found to be greater than female tick. Four genera of hard ticks were encountered. From identified genera, *Ambylomma* 220 (32.2%) was the most abundant genus followed by the genus *Rhipicephalus (Boophilus)* 204 (30.0%), whereas the most minor proportion of hard tick genera was recorded in *Hyalomma* (116 (16.8%; Table 2).

### 3.3. Distribution of Ixodid Ticks Genera on Different Body Arts of Study Animals

*Amblyomma*, *Rhipicephalus (Boophilus)*, and *Rhipicephalus* appeared to be dominant on the dewlap body part of the animal, whereas *Hyalomma* ticks more prefer the sternum of the animal (Table 3). Moreover, higher number of hard tick genera was counted in sternum (203) followed by dewlap (178), whereas fair distribution was observed in udder, scrotum, and other body parts.

## 4. Discussion

In the present study, high prevalence of ticks (71.6%) was registered (Table 1). The prevalence of the present finding is in line with higher prevalence report obtained from different part of the country [15] (70.31%) in Bishoftu town, Ethiopia, [16] (74%) in Bahir Dar, Ethiopia, [17] (65.5%) in Soddozuria districts, Wolaita Zone, Ethiopia, and [16] (68.12%) in the high land of Decha Woreda of Keffa Zone, Ethiopia. But, the present finding is relatively lower than that of previous study [18] (97.8%) and [19] (82%) in Bedele, Oromiyia, [20] (81.25) in southwest Ethiopia, and [21] (91.50%) in Gesha districts of Southern Ethiopia. On other hand, our study is higher than the finding reported by Tiki and Addis [22], with a prevalence of 25.64% in Holeta town, Ethipoia and [23] (38%) in the Chiro District, West Hararghe Zone, East Oromiya. The inconsistency among these studies may be attributed to variation agro ecology, animal health practice and sample size, and method of detection with in their respective study area.

In our finding, *Ambylomma* was the most dominant tick genera, accounting for (32.2%) of the total proportion. This finding is agree with the previous study [14] (34.9%) in Arbegona District, Southern Ethiopia and [24] (43.46%) in Humbo district, SNNPR of Ethiopia, indicated *Amblyomma* as the leading tick genera. This finding was in contrast to the previous report by Fesseha and Mathewos [8] in Hosana District, Hadiya Zone, Southern Ethiopia and [25] in Arsi Zone, Oromia Region, indicated *Hyalomma* and *Rhipicephalus (Boophilus)* were the most dominant tick genera, respectively. The difference could be due to the variation in the season during which the sample collection and agro ecology of the study conducted. The area of tick predilection in this study was dewlap, sternum, scrotum, udder, and other body parts (ear, head, tail, anal, and leg). The results showed that various tick genera have relatively different predilection sites and agree with [26] report of hard tick's infestation in cattle in and around Honkola Wabe District. With regard to distribution pattern of ticks, *Amblyomma* and *Rhipicephalus* (*Boophilus*) had relatively distribution to more on dewlap followed by sternum body regions of animals, which is in line with the work of Nateneal et al. [19]. In this study, *Rhipicephalus* ticks more preferred in other body parts (tail, anal, ear, and leg), which also agree with the findings of Kassa and Yalew [27], in and around Haramaya District, Eastern Ethiopia.

There was variation in age-wise prevalence with higher prevalence was recorded in older cattle (82.3%) followed by adults (72.9%), and the least prevalence was registered in young age cattle with a prevalence of (59.8%) and the difference found statically significant (*P* < 0.05). This result agrees with the report of Kemal et al. [14] with a highest prevalence of 98.4% in older age of cattle in Arbegona District, Southern Ethiopia. The higher tick burden in older cattle may be associated with low immunity and resistance, whereas young cattle were kept in doors that had less chance of acquiring the tick infestation and had better maternal immunity. In contrast to study, there are previous report by Shichibi et al. [21], in Saylem, Gesha, and Masha Districts, Southern Ethiopia with prevalence higher in adult and Yalew et al. [28], around Haramaya town, Eastern Hararghe, Ethiopia with higher prevalence in young cattle than another age group. This may be due to variation sampling distribution, sample size, and method of detection.

There was variation in sex-based prevalence with higher prevalence recorded in males (78.3%) compared with females (65.5%). This finding agrees with the finding of Chumburo and Bayou [29], who reported prevalence rate of tick infestation was found to be higher in male cattle (42.2%) than females (34.7%) and Hordofa et al. [9], who reported the prevalence of tick infestation was higher in male cattle (100%) than females (46.3%) in and around Honkola Wabe District. On the other hand, this finding was disagree with the previous findings of Shichibi et al. [21], who reported higher prevalence in female (89.77%) and Nateneal et al. [19], who indicated the higher tick prevalence in female (52.9%) than male (41%). Therefore, the variation in tick infestation of male and female cattle in the current research might be attributed to differences in management systems, sample size, and sampling distribution.

In this study, the tick burden was higher in cattle with poor body condition (87.2%) followed by medium body condition (69.4.2%). This finding agree with the works of Wolde and Mohamed [17], Nateneal et al. [19], and Hordofa et al. [26], who reported cattle with poor body condition were significantly (*P* < 0.05) infested more than that of cattle with normal body condition. This was also comparable with the finding of Kemal et al. [14], who reported that higher tick infestation in poor body condition of cattle than any other groups. This may be because poorly conditioned animals had low resistance to tick infestation and lack enough body capacity to build resistance, whereas animals with good body condition showed reasonable combat to the infestation (Manan, 2007). Moreover, a higher tick burden might be a cause for poor body condition.

## 5. Conclusion

The most important genera investigated in the study area were *Rhipicephalus (Boophilus)*, *Ambylomma*, *Hayloma*, and *Rhipicephalus*. The distribution of tick genera in this study indicated that there is high burden of ticks in the area. The distribution of ticks is not fixed, but it is determined by a complex interaction of factors, such as climate, host density, host susceptibility, grazing habits, and pasture–herd management. The study demonstrated that tick infestation was the major challenge in reducing productivity and cause health problems of cattle in the Areka District with an overall prevalence of 71.6% revealing ticks is common and important ecto-parasite of cattle. A tick has a great production and economic impact by causing, decreasing milk and meat yield and quality, and extra expense for treatment and health care. It also decreases the quality of skin and hides in the tannery industries. Poor management systems and lack of awareness about ticks were factors in the distribution of ticks in the areas. Therefore, the author has recommended that good management practice like regular deworming using acaricides should be practiced and it is also important to create awareness among livestock owners about the veterinary importance of ticks for integrated tick control.

## Figures and Tables

**Table 1:** Potential risk factors for ixodid ticks infestation status of cattle in the case of Areka District.

Risk factors		No. of animals examined	No. of positive animals	*χ* ^2^	*P*-Value	COR (95% CI)
Kebeles	Woyibo	47	25 (53.2%)	—	—	Ref.
	Korke	67	53 (79.1%)	10.2	0.04	3.3 (1.46–7.57)
	Adibinch	76	57 (75.0%)			2.6 (1.22–5.72)
	Legama	57	41 (71.9%)			2.3 (0.99–5.08)
	Tokiso	137	99 (72.3%)			2.4 (1.16–4.55)
Sex	Female	200	131 (65.5%)	—	—	Ref.
	Male	184	144 (78.3%)	7.7	0.006	1.9 (1.20–2.99)
Age	Young	127	76 (59.8%)	—	—	Ref.
	Adult	133	97 (72.9%)	15.7	0.001	1.7 (0.945–3.132)
	Old	124	102 (82.3%)			3.1 (1.74–5.56)
Body condition	Good	127	73 (57.5%)	—	—	Ref.
	Medium	124	86 (69.4%)	28.7	0.001	0.6 (0.35–1.01)
	Poor	133	116 (87.2%)			3.0 (1.59–5.69)

**Table 2:** Distribution and sex ratio of adult ixodid ticks genera identified in the Areka District.

Hard tick genera	Proportion	Sex		
		Male	Female	M : F ratio
*Ambylomma*	220 (32.2%)	169	51	3.3 : 1
*Rhipicephalus (Boophilus)*	204 (30.0%)	174	30	5.8 : 1
*Rhipicephalus*	143 (21%)	97	46	2.1 : 1
*Hyalomma*	116 (16.8%)	71	45	1.6 : 1
Total	683 (100%)	511	172	2.9 : 1

**Table 3:** Genera of ixodid ticks and their distribution on body regions of cattle in the Areka District.

Body region	*Ambylomma* (counted)	*Rhipicephalus* (*Boophilus*) (counted)	*Rhipicephalus* (counted)	*Hyalomma* (counted)	Total
Dewlap	73	70	5	30	178
Udder	37	26	24	17	104
Scrotum	33	32	12	18	95
Sternum	66	62	26	49	203
Others	11	14	76	2	103
Total	220	204	143	116	683

## Data Availability

Data supporting this research article are available from the corresponding author or first author on reasonable request.
